# Precision minimally invasive strategy for Hirschsprung’s disease: from anatomical resection to whole-cycle functional management—a review

**DOI:** 10.3389/fped.2026.1916809

**Published:** 2026-07-30

**Authors:** Kai Zheng, Wenyue Ma, Rui Li, Daqing Sun

**Affiliations:** Department of Pediatric Surgery, Tianjin Medical University General Hospital, Tianjin, TJ, China

**Keywords:** biofeedback training, defecation function, hirschsprung's disease, minimally invasive surgery, risk stratification

## Abstract

**Background:**

Minimally invasive radical surgery for Hirschsprung's disease (HD) has achieved substantial advances in anatomical accuracy and perioperative safety, yet postoperative defecation dysfunction remains a key factor limiting long-term quality of life in pediatric patients. Existing reviews seldom elaborate the integrated whole-cycle functional management paradigm.

**Objective:**

To summarize available study on precision minimally invasive strategies for HD and propose a whole-cycle functional management framework.

**Methods:**

This review summarizes information from major databases, including ScienceDirect, Google Scholar, Scopus, PubMed and Springer Link. Publications were selected without date restrictions and and used terms such as Hirschsprung's disease, minimally invasive surgery, defecation function, risk stratification, biofeedback training; non-English literature, conference abstracts without full text were excluded.

**Results and conclusions:**

In this review, we summarized current minimally invasive, precision therapeutic strategies for Hirschsprung disease. Additionally, we analysed the corresponding comprehensive care pathway, to highlight a paradigm shift—from isolated anatomical resection to holistic, function-oriented management across the entire disease trajectory.

## 1 Introduction

The core goals of radical surgery for Hirschsprung's disease (HD) include complete resection of aganglionic bowel segments, relief of intestinal obstruction, and ensuring perioperative safety, with surgical success primarily measured by anatomical indicators such as absence of anastomotic leakage and residual lesions (1). Continuous iteration of surgical techniques, from open surgery to laparoscopic and transanal approaches, has achieved higher anatomical precision and less surgical trauma, provides an objective anatomical basis for postoperative functional recovery. Minimally invasive approaches—particularly laparoscopic-assisted and purely transanal techniques—have become standard in HD radical resection (2). Aiming at anatomical precision and miniaturized incisions, these techniques reduce surgical trauma and hospital stay. For example, new minimally invasive techniques such as transanal endoscopic myotomy (TEM), which are based on the principle of “third space” endoscopy, have provided new options for treatment (3). Although precise minimally invasive surgical techniques for HD continue to advance, postoperative bowel dysfunction can also compromised long-term quality of life in affected children (4). Postoperative defecation dysfunction presents not only as fecal incontinence but also frequently as refractory constipation or dyschezia. These problems seriously affect the development of children's physical, psychological and social functions. For example, a retrospective study of children with HD after radical surgery found that female patients with HD had a higher incidence of defecation dysfunction and a lower health-related quality of life score (5). However, some long-term series report acceptable continence rates [e.g., 72% of children experienced improvements in bowel movement frequency (6)], suggesting that the relationship between anatomical precision and functional outcome is not linear. Patient selection, surgical technique variations, and follow-up methodology contribute to this heterogeneity.

Whole-cycle functional management refers to a whole-course diagnosis and treatment system covering preoperative functional assessment and risk stratification, intraoperative precise protection of functional structures, postoperative structured rehabilitation and long-term functional follow-up. The primary objective is to optimize the restoration of defecatory function and enhance patients' quality of life. A risk stratification framework centered on accurate preoperative functional assessment should incorporate precise intraoperative positioning guided by functional anatomical landmarks. Some scholars have proposed the anorectal line (ARL) as an alternative anatomical landmark to the more subjective dentate line (DL), which theoretically minimizes injury to the anal transition zone to sensory innervation and improve postoperative bowel function (7). The ARL is preferred because reliance on the DL poses a risk of injuring the sensory innervation within the anal transition zone, which is critical for normal defecation. Preserving an intact anal transition zone and performing longitudinal posterior rectal cuff myotomy for release are essential anatomical foundations for maintaining normal defecation function. However, the anatomical consistency and clinical value of this landmark lack validation from multicenter large-sample studies and have not become a universal clinical standard. Objective assessment of postoperative bowel function is critical for effective long-term monitoring and the early detection of complications.

Existing reviews on HD mostly focus on technical dimensions such as surgical approaches or postoperative complications, and a systematic elaboration of the whole-cycle precision management paradigm integrating preoperative diagnosis, intraoperative minimally invasive protection and postoperative functional rehabilitation is still lacking. Preoperative assessment systems are mostly scattered in single dimensions such as anatomy, pathology and function, lacking an individualized risk stratification framework integrating multimodal information, which cannot accurately predict postoperative functional outcomes. Localization techniques such as intraoperative frozen pathology and ICG fluorescence are mostly applied alone, and the operational specifications and decision-making paths for their synergistic application have not yet formed standardized schemes. Postoperative rehabilitation is mostly passive intervention after complications, lacking active stratified rehabilitation paths based on preoperative risk stratification and matching surgical methods, making it difficult to translate the anatomical advantages of minimally invasive techniques into functional benefits. This review summarizes the evidence system of precision minimally invasive technology for HD, constructs a whole-cycle functional management paradigm, and proposes future research directions, so as to provide evidence-based reference for clinical practice.

## 2 Literature search and selection strategy

This work is a narrative review aiming to integrate current clinical evidence and propose a conceptual framework for whole-cycle functional management of HD, rather than quantitative pooled analysis of effect sizes. To ensure methodological transparency and minimize selection bias, a standardized literature retrieval and screening process was implemented. A systematic literature search was conducted across five electronic databases: ScienceDirect, Google Scholar, Scopus, PubMed, and Springer Link, with no restriction on publication year (final search update: June 2026). The search strategy combined medical subject headings (MeSH) and free-text terms, including: (1) disease terms: “Hirschsprung's disease” OR “HD”; (2) intervention terms: “minimally invasive surgery” OR “laparoscopic surgery” OR “robotic surgery” OR “transanal pull-through”; (3) outcome terms: “defecation function” OR “fecal incontinence” OR “constipation” OR “bowel function” OR “quality of life”; (4) management terms: “risk stratification” OR “biofeedback” OR “rehabilitation” OR “perioperative management”.

Inclusion criteria: (1) peer-reviewed original clinical studies (prospective/retrospective cohorts, randomized controlled trials); (2) systematic reviews and meta-analyses focusing on HD surgical treatment or functional outcomes; (3) international clinical guidelines and consensus statements on HD management; (4) full-text publications in English. Exclusion criteria: (1) non-English literature; (2) conference abstracts without available full tex.

The study selection was performed independently by two researchers. First, titles and abstracts were screened to exclude obviously irrelevant literature. Then full texts were retrieved for further assessment according to the eligibility criteria. Any discrepancies between the two researchers were resolved through consultation with a third senior pediatric surgeon to reach a consensus.

## 3 Current status and challenges of minimally invasive techniques in radical resection of HD

The widespread adoption of laparoscopic and robot-assisted surgery represents a paradigm shift of HD radical resection from traditional open surgery to minimally invasive precision surgery. Laparoscopy enhances the accuracy of transition zone identification and lesion resection, while robotic surgery offers unique advantages for fine dissection in the narrow pediatric pelvic cavity, both collectively improving anatomical precision and reducing surgical trauma (8). Compared with open procedures, minimally invasive techniques significantly reduce intraoperative trauma, postoperative pain and incision-related complications, shorten the recovery time of intestinal function and length of hospital stay. More importantly, the magnified visual field allows surgeons to more clearly identify the pelvic nerve plexus and anal sphincter structure, which provides an anatomical basis for better protection of defecation and urinary function (9). Compared with open procedures, minimally invasive techniques significantly reduce intraoperative trauma, postoperative pain and incision-related complications, shorten the recovery time of intestinal function and length of hospital stay. More importantly, the magnified visual field allows surgeons to more clearly identify the pelvic nerve plexus and anal sphincter structure, which provides an anatomical basis for better protection of defecation and urinary function. Although minimally invasive surgery is technically more demanding and has a longer learning curve, its advantages in reducing postoperative adhesive intestinal obstruction and the need for enterostomy have gradually become a consensus.

During preoperative assessment, it is essential not only to evaluate imaging studies and histopathological findings but also to holistically integrate patient-specific risk factors. Studies have shown that female sex is an independent risk factor for postoperative complications within 30 days (OR = 5.065, *P* = 0.024) and long-term defecation dysfunction (OR = 3.153, *P* = 0.019) in HD (10). Therefore, special attention should be paid to gender differences in preoperative evaluation. More detailed nutritional optimization programs and perioperative management plans should be developed for female patients. In addition, children with chromosomal abnormalities and multiple congenital malformations (such as trisomy 21) often have poorer physiological reserve and lower surgical tolerance. Clinicians need to comprehensively evaluate their general status before surgery and develop targeted postoperative monitoring and rehabilitation protocols (11). Large-sample long-term functional outcome studies on children with HD complicated with trisomy 21 are still scarce, representing an important content to be supplemented in the future risk stratification system. Other conditions—such as long-segment HD or associated congenital malformations (e.g., total colonic aganglionosis, which is prevalent in Waardenburg syndrome type IV)—may indicate a more complex pathophysiology and a less favorable functional prognosis (12). The pooled prevalence of preterm infants with HD ranged from 5.0% to 5.6%. The pooled incidence of constipation in HD patients was 44%, and that of enterocolitis was 13% (13). This suggests that clinicians need to evaluate the physiological reserve and surgical tolerance of special children more carefully before operation, and develop more targeted postoperative monitoring programs. The evaluation of whole-cycle functional management is helpful to identify the risk factors for postoperative poor function.

Postoperative management emphasizes the multidisciplinary team (MDT), including the participation of pediatric surgeons, gastroenterologists, nutritionists, rehabilitation physicians and psychologists. Studies have shown that postoperative management needs to focus on the prevention of Hirschsprung-associated enterocolitis. The imbalance of intestinal flora, especially the increase in the abundance of Proteobacteria, is a key factor in the occurrence of HAEC (14). Therefore, probiotic intervention or fecal microbiota transplantation may be considered to regulate the intestinal microecology after surgery to reduce the risk of HAEC. Finally, the goal of whole-cycle functional management is to take the long-term quality of life of patients as the ultimate concern (15). A large number of studies have confirmed that postoperative defecation function is the most critical factor affecting the quality of life of patients. The long-term defecation function of children with total colonic aganglionosis can gradually improve with age, but there are differences in the perception of quality of life between children and caregivers. Clinical follow-up needs to pay attention to children's subjective feelings and psychological status (16). Meanwhile, easy-to-understand health education materials should be developed to improve families' participation in postoperative rehabilitation management and ensure the home implementation effect of rehabilitation protocols (17). Therefore, clinical management of functional rehabilitation should not only address the physiological aspects of defecation but also incorporate psychological support, social reintegration, and long-term follow-up to optimize patients' physical and mental well-being and social functioning.

## 4 Key techniques and applications of preoperative functional assessment

The substantial heterogeneity in postoperative defecation outcomes among HD patients is closely related to multiple factors including lesion length, comorbidities, sex, and surgical technique. Preoperative functional assessment and risk stratification are the prerequisites for reducing outcome heterogeneity and realizing individualized management. Anorectal manometry (ARM) is the cornerstone of objective functional assessment for HD, with core clinical value in preoperative baseline evaluation, postoperative efficacy monitoring and complication identification (18). Among them, Resting Anal Pressure (RAP) mainly reflects the functional state of the internal sphincter, which is the basic index to evaluate the anal self-control ability. The recovery of RAP after radical resection of HD is strongly correlated with the incidence of postoperative soiling and incontinence. For instance, a comparative study of transumbilical and anal laparoscopic pull-through (HTALP) vs. total transanal laparoscopic-assisted pull-through (TTLAP) for common-type HD revealed that anal resting pressure was significantly lower in the HTALP group than in the TTLAP group at 1 and 3 months postoperatively, suggesting that HTALP causes less damage to the anal sphincter. Variations in the extent of sphincter injury influence early fecal control (19). Another long-term follow-up study of the common type of HD also confirmed that anal resting pressure was consistently lower in patients who underwent total transanal laparoscopic pull-through (TTLP) than in those who underwent transanal simple pull-through (PTEP) from 1 to 10 years after surgery (20). In addition, the presence or absence of Rectoanal Inhibitory Reflex (RAIR) is a key indicator to evaluate the integrity of the rectal sensory and nerve reflex pathway. The absence of RAIR is one of the typical diagnostic features of HD, and the recovery of RAIR after surgery is regarded as a sign of good neurological reconstruction. A study involving patients with anorectal malformations and HD demonstrated that all seven subjects exhibited an absent anorectal inhibitory reflex (RAIR) on preoperative anorectal manometry. In healthy infants and young children, about 16% of subjects have negative RAIR but normal defecation function during follow-up, suggesting that negative RAIR may not represent a pathological condition in a specific age or individual and needs to be judged in combination with clinical judgment (21).

In order to ensure the accuracy and comparability of manometry results, it is essential to establish standardized operating procedures and data interpretation norms. At present, High-Resolution Manometry (HRM) and 3D High-Definition Anorectal Manometry (3D-HDAM) have gradually replaced the traditional water perfusion manometry. It provides finer spatial and temporal resolution. High-resolution manometry (HRM) and 3D high-definition anorectal manometry (3D-HDAM) have been increasingly adopted, providing finer spatiotemporal resolution (22, 23). The new air-filled catheter manometry has more advantages in operational convenience, patient tolerance and detection efficiency, promoting the clinical popularization of manometry technology (24). In the interpretation of the data, the influence of physiological factors such as age and gender must be considered. Data from studies in healthy children indicate that anal canal function parameters (e.g., HPZ, resting pressure, compression pressure) gradually increase with age, which emphasizes the importance of using age-matched normal values as a reference when assessing postoperative function in children, especially infants (25). In recent years, deep learning models can effectively process complex spatio-temporal information. These advances indicate that artificial intelligence-based manometry is expected to achieve more objective, accurate and standardized functional assessment. However, other studies have noted that the correlation between digital rectal examination (DRE) and objective manometry methods such as 3D-HDAM and surface electromyography is not ideal, especially when assessing resting anal pressure (26). This suggests that although manometry provides quantitative data, clinicians still need to combine DRE and other physical examination results to make a comprehensive judgment and avoid over-reliance on a single technique. Therefore, the standardized procedure should clarify the steps of patient preparation (such as bowel preparation and body position), catheter insertion and positioning, parameter acquisition (including resting, squeezing, simulated defecation and other stages) and quality control. The data interpretation needs to integrate multi-parameter information, refer to age- and sex-matched normal values, and finally make a comprehensive assessment in combination with clinical symptoms.

Colonic transit test (CTT) is the main method to objectively evaluate colonic motility, which can distinguish slow-transit constipation from normal-transit constipation and provide a basis for the etiology judgment of postoperative defecation disorders (27). In patients with HD after radical resection, the evaluation of colonic transit function is particularly important, because postoperative abnormal innervation or changes in intestinal anatomy often lead to dysfunction of colonic transit function (28). Therefore, CTT is not only an objective tool to assess colonic dynamics, but also an effective means to monitor disease progression and response to treatment. In terms of preoperative risk stratification and surgical plan optimization, the value of colonic transit test has become increasingly prominent for patients undergoing radical resection of HD. The bedside “Bananagram” assessment for children with colostomy can non-invasively evaluate distal colonic transit function. An ejection diameter <0.3 cm indicates poor transit function and predicts the risk of postoperative reoperation, providing an intuitive basis for preoperative surgical plan selection (29). This simple, radiation-free bedside assessment method provides a visual basis for preoperative judgment of colonic function, thereby guiding the choice of surgical timing and method. The inclusion of colonic transit test in the routine preoperative evaluation system of HD can help surgeons more accurately predict postoperative defecation function, so as to formulate individualized surgical plans, such as choosing a more appropriate anastomosis method or considering intestinal management intervention at the same time, to reduce the risk of postoperative constipation or soiling.

Anatomical imaging is indispensable components of the preoperative multi-dimensional assessment system for HD, providing morphological basis for lesion localization and complementing functional and genetic evaluation. Barium enema is the most widely used preoperative screening and anatomical localization tool in clinical practice. It can intuitively display the morphological characteristics of the colon, identify the transition zone between the dilated proximal bowel and the narrow distal bowel, and preliminarily classify the clinical subtype (short-segment, long-segment, or total colonic aganglionosis), which provides important reference for surgical approach selection and preoperative risk stratification (30). However, barium enema has limited sensitivity in neonates and young infants due to the unobvious transition zone in early stage, and it can only provide morphological information but cannot reflect the innervation status and functional level of the intestine (31). Therefore, it needs to be interpreted in combination with anorectal manometry and pathological biopsy.

RET is the central causative gene in HD, encoding a receptor tyrosine kinase essential for the migration, proliferation, differentiation, and survival of the enteric neural crest cells (32). Other key genes—including SOX10—also contribute to HD pathogenesis by participating in the complex signaling network governing neural crest development (33). Targeted next-generation sequencing (NGS) panels have become a vital tool for preoperative genetic evaluation in HD, simultaneously analyzing coding regions and critical non-coding regulatory elements of known disease-associated genes, thereby significantly improving mutation detection rates (34). A whole-exome sequencing study of 301 HD probands and 109 core families identified 19 risk genes enriched for ultra-rare pathogenic coding variants, explaining 17.5% of population-attributable risk, of which 6.5% is accounted for by newly implicated candidate genes (35). Its identification is crucial for multidisciplinary management and prognostication. Genetic testing plays an indispensable role in distinguishing syndromic forms, clarifying etiology, predicting comorbidities, and guiding familial counseling. Recent genetic studies using NGS approaches, such as whole exome sequencing and whole genome sequencing (WGS), involving tens of severe HSCR trios (proband and unaffected parents) and >150 sporadic HSCR cases have identified a dozen new HSCR candidate genes (36). A polygenic risk model computed based on genetic data of 190 European HD patients and 740 controls suggested that the risk of HD with both rare and common variants is collectively larger than that with only rare coding variants or only common regulatory variants (37). Using the polygenic risk score (PRS) aggregating the genetic risk of many genetic variants with low penetrance can help recover the missing heritability of HD (35). The use of PRS in a clinical setting is still immature, although targeted NGS panels represent a valuable first-tier diagnostic tool—supporting preoperative diagnosis, genetic counseling, and surgical planning—their inherent limitations underscore the necessity of complementary, more comprehensive genomic approaches, such as whole-genome sequencing, in future diagnostic frameworks (Table 1).

**Table 1:** Three-tier preoperative risk stratification and matched management recommendations for HD.

Risk tier	Key inclusion features	Recommended management
Low risk	Short/common-segment HD; no major comorbidities; preserved baseline anal function	Single-stage transanal pull-through; routine postoperative follow-up
Intermediate risk	Long-segment HD; mild congenital malformations; abnormal colonic transit	Laparoscopically assisted pull-through; enhanced pelvic nerve protection; early rehabilitation intervention
High risk	Total colonic aganglionosis; chromosomal abnormalities/multiple malformations; preterm low birth weight	Staged surgery; multidisciplinary supportive care; long-term regular follow-up

## 5 The progress and application of intraoperative precise positioning technology

In the radical resection of HD, accurate identification and resection of the aganglionic bowel segment while maximizing the preservation of normal bowel are key to determining the success of the operation and the recovery of postoperative defecation function. The “transition zone” is mainly determined by observing the diameter, peristalsis and vascular morphology of the intestine. Rapid and reliable pathological techniques are the basis of intestinal identification (38). The conventional clinical principle requires the anastomosis to be placed in normal ganglionic bowel proximal to the transition zone to avoid postoperative constipation and obstruction caused by transition zone pull-through. However, there is no unified standard for the specific proximal extended resection range, and most centers routinely extend 1–3 cm (39). A single-center retrospective study of 87 children in 2024 explored the value of resection range, showing no significant difference in the incidence of postoperative enterocolitis or reoperation between children with extended proximal margin (EPM) ≤5 cm and >5 cm, but the short resection group had a higher frequency of involuntary daytime bowel movements. This result suggests that excessively extending the resection range cannot reduce the risk of long-term obstruction and enterocolitis, but may affect defecation control function by losing too much stool-storing bowel (40). However, due to the small sample size and confounding bias of the retrospective design, this conclusion still needs verification with higher-level evidence. Clinical decisions should be made individually based on the patient's lesion type and preoperative functional status.

Intraoperative frozen section(FS) technology is the most widely used and the core “gold standard” auxiliary method. By rapidly freezing, sectioning and staining the intestinal wall tissue obtained during the operation, this technique can provide pathological diagnosis and determine the presence or absence of ganglion cells (41). A single-center study from Indonesia showed that, with paraffin permanent section as the gold standard, the accuracy, sensitivity and specificity of intraoperative frozen section were 88%, 95% and 92.8% respectively, which was numerically higher than the 82% accuracy of barium enema (42). However, the two examinations have different clinical positions: barium enema is used for preoperative screening and localization, while frozen section is the gold standard for intraoperative determination of resection margin, and direct comparison of accuracy is of limited clinical significance. Another study from 2023 provided a new perspective on the pathological grading of ganglion cells in 357 children. A lower grade was found to be significantly associated with better postoperative bowel function and a lower incidence of enterocolitis (43). This suggests that intraoperative pathological evaluation should not only be limited to the “presence or absence” of ganglion cells, but also assess their morphological quality, which provides a basis for individualized and precise decision making of the extent of resection. Intraoperative frozen pathology has two inherent limitations. First, ganglion cells in neonates and infants are not yet fully mature, with sparse cell distribution in the transition zone, so the diagnostic accuracy is highly dependent on the pathologist's experience and specimen quality. Second, it can only provide local information of sampling sites, and the resection boundary needs to be dynamically determined by combining preoperative imaging and intraoperative multi-point stepwise biopsy. Although this mode of “multi-point biopsy + real-time feedback” increases the operation time, it greatly improves the accuracy of the operation. It is the ideal technical path to achieve “whole-cycle functional management” bowel resection.

Indocyanine green (ICG) is a water-soluble near-infrared fluorescent dye. Its biological characteristics make it an ideal tracer for intraoperative angiography. ICG is used to evaluate anastomotic perfusion in colorectal surgery and is practical for evaluating intestinal perfusion in pediatric gastrointestinal surgery. No severe adverse events such as anaphylactic shock have been reported in pediatric gastrointestinal surgery, and the overall safety profile is favorable (44). Indocyanine green fluorescence angiography (ICG-FA) can be used to visualize tissue through an infrared camera. This capacity to evaluate tissue perfusion enables ICG fluorescence technology to surpass the limitations of traditional visual assessment, providing surgeons with objective and dynamic blood flow data (45). During radical resection for HD, surgeons traditionally assess bowel viability using subjective indicators such as color, peristalsis, and marginal artery pulses. However, they now also evaluate blood perfusion and tissue activity intraoperatively. By displaying the fluorescence intensity of the bowel wall and its time-intensity curve in real time, ICG-FA allows surgeons to accurately identify areas of poor perfusion (46). Furthermore, the colonic inversion technique itself prevents duodenal compression and ensures a tension-free anastomosis by preserving marginal arterial branches and employing other specific maneuvers. ICG-FA is utilized specifically to map the blood supply of the pulled-through colon intraoperatively (47). A retrospective study on surgical outcomes in HD found that the use of ICG quantitative perfusion analysis (Fmax > 30 AU, Tmax < 30 s) to guide anastomotic site selection significantly reduced postoperative anastomotic inflammation at 5–7 days postoperatively and resulted in a higher proportion of patients remaining free of HAEC (78.1% vs. 56.8%) (48). Although findings regarding whether ICG-FA significantly reduces major anastomotic complications (e.g., leakage) in the surgical management of HD remain inconsistent, current evidence strongly supports its efficacy in reducing anastomotic inflammation, shortening the length of hospital stay, and lowering HAEC incidence. The quantitative parameters of ICG-FA can also more precisely predict tissue activity, thereby improving the accuracy of surgery. The use of ICG-FA may enable surgeons to maximize preservation of viable bowel segments while ensuring anastomotic safety. A prospective clinical trial in pediatric bowel resection found that the results of ICG-FA assessment differed from the surgeon's judgment based on traditional methods in 62% of cases (49). Laparoscopic-assisted colonic rotation for patients with HD utilized intraoperative ICG-FA to verify adequate colonic perfusion, preserve marginal artery supply, and prevent duodenal compression. The anastomosis was tension-free, associated with minimal complications, and proved cost-effective (50, 51). Studies indicate that a clear ICG perfusion signal can guide surgeons to select a well-perfused anastomotic level, potentially reducing the risk of anastomotic leakage. If the target bowel exhibits homogeneous, bright fluorescence, perfusion is sufficient to proceed with a safe anastomosis. If the signal is dim, delayed, or patchy, indicating poor perfusion, the resection level should be promptly adjusted until satisfactory perfusion is achieved. ICG assessment is qualitative/semi-quantitative and lacks a universal threshold for optimal perfusion. Thus, ICG can only evaluate intestinal perfusion status and cannot judge intestinal innervation, so it cannot replace the gold standard status of intraoperative frozen section. Clinical application requires comprehensive judgment combined with the surgeon's experience (Table 2). Current evidence for ICG application in HD is mainly based on single-center experience, and high-quality randomized controlled trials are lacking to confirm its ability to reduce the incidence of serious complications such as anastomotic leakage and stenosis.

**Table 2:** Comparison of information density and decision-making value of intraoperative localization technologies.

Dimension	FS	ICG-FA
Information type	Histological: Determines presence/absence and maturity of ganglion cells. Provides point-specific, static cellular-level data.	Physiological: Assesses real-time bowel perfusion. Provides holistic, dynamic functional data.
Data quantification	Primarily qualitative: Positive/negative interpretation with 88% accuracy. Though ganglion cell grading exists, no widely accepted quantitative threshold (42).	Well-defined quantitative thresholds: Recommended safe anastomosis criteria: Fmax > 30 AU, Tmax < 30 s, offering objective, reproducible cutoffs (48).
Decision impact	Defines resection margin: Determines the proximal extent of aganglionic segment.	Modifies surgical strategy: Alters anastomotic level or approach, influencing 62% of cases (49).
Key limitation	Cannot assess blood supply: May lead to resection of viable (ganglionic) but poorly perfused bowel, increasing anastomotic complications.	Cannot assess innervation: May preserve well-perfused but aganglionic bowel, leading to poor functional outcomes.
Synergistic advantage	The complementary nature of FS and ICG-FA suggests a theoretical dual-modal strategy: FS confirms the presence of ganglion cells to ensure radical resection, while ICG-FA evaluates bowel perfusion to ensure anastomotic safety. This combined approach may reduce both residual aganglionic bowel and anastomotic complications, but to date, no clinical studies have directly validated its efficacy or safety in HD surgery. It remains a promising direction for future prospective research.	

The optimization of surgical approach and procedural details is also an important part of the intraoperative functional protection strategy. With three-dimensional high-definition vision and the flexibility of wrist instruments, robot-assisted surgery can perform dissection at the level close to the longitudinal rectal muscle sheath, maximally preserving the sphincter and perirectal nerve plexus and reducing intraoperative functional structure injury. Compared with pure transanal surgery, laparoscopic-assisted approach provides a clearer pelvic view and reduces the risk of accidental sphincter injury caused by blind operation, providing an anatomical basis for postoperative functional recovery. A study utilized statistical software implemented in Python to compare hospital costs and surgical outcomes. Results showed that children with Hirschsprung disease and prolonged operative times derived significantly greater benefit from robotic-assisted laparoscopic surgery (RALS). While RALS entails higher inpatient costs compared to conventional laparoscopic surgery (LS), it is associated with superior postoperative outcomes and proves to be cost-effective (52). In a 2023 multicenter prospective study involving 156 children with rectosigmoid HD, robot-assisted rectosigmoid resection (RAPS) was performed, with preservation of the sphincter and perirectal nerves achieved by dissection close to the epimysial muscle of the longitudinal rectum. The results showed that the postoperative defecation function score of children aged 4 years and older was 17.32 ± 2.63, and 90.91% of the children reached a moderate-good level. The postoperative defecation control score (POFC) showed an increasing trend with age (53). Robotic surgery may improve the prognosis in older children, delayed diagnosis or complex cases requiring reoperation due to superior visualization of the pelvic anatomy (54). A Bayesian network meta-analysis comparing four procedures showed that laparoscope-assisted TAPT was superior to OD, LD and TEPT in reducing intraoperative bleeding, improving gastrointestinal function, and reducing postoperative incidence of fecal soiling, constipation and HAEC (55).

The aganglionic segment is typically confined to the rectosigmoid region, rendering an endoscopic approach a logical consideration in HD (56). Its length is estimated via serial rectal biopsies taken at 2–3 cm intervals (57). The procedure involves a mucosal incision, submucosal tunneling to the distal margin of the aganglionic segment, followed by a myotomy and mucosal closure using endoclips. Myotomy near the anal verge requires caution to avoid injury to the external anal sphincter (58). Submucosal endoscopy—termed per-rectal endoscopic myotomy (PREM)—has been performed in nine HD cases (59). The mean length of the aganglionic segment was 6.3 cm. All procedures were successful, with a mean duration of 96 min and no major intraoperative adverse events. At a median follow-up of 17 months (range: 9–58 months), stool frequency improved and laxative use decreased in all patients (59). Beyond this small series, evidence supporting PREM in HD remains limited. Large-scale, long-term studies are needed before PREM can be recommended for routine clinical use.

Therefore, the comprehensive application of accurate and rapid pathological characterization, objective perfusion evaluation of ICG, and fine anatomy of minimally invasive surgery constitute the three pillars of modern intraoperative functional protection strategy in HD.

## 6 Construction of postoperative structured rehabilitation management system

Due to the congenital loss of enteric ganglion cells and the potential effect of surgery on the anal sphincter and pelvic floor muscles, children with HD often face problems such as dysesthesia of defecation, sphincter dyssynergia and fecal incontinence. Biofeedback training is designed to address these core dysfunctions. A randomized controlled trial of postoperative children with HD showed that electrical stimulation with either rectal or surface electrodes combined with routine anal sphincter exercise and biofeedback training resulted in significant improvement in bowel function scores (effect size d = 1.21). And there was a significant decrease in the fecal incontinence Score (effect size d as high as 1.15) (60). The study further showed that small but significant improvements in all measures remained from the end of treatment to follow-up, suggesting that the effects of biofeedback training were sustained. A study reported on biofeedback therapy in a 17-year-old male with HD and total colonic aganglionosis, who underwent a Duhamel pull-through in infancy and later developed recurrent fecal soiling and constipation. The clinical examination showed stool retention with preserved perianal sensation and intact anal sphincter tone—consistent with retentive fecal incontinence. After eight biofeedback sessions, his Revised Fecal Incontinence Score improved from 16/20 to 1/20, resulting in complete symptom resolution (61). However, these mechanisms are mostly extrapolated from studies of functional defecation disorders, and specific evidence in HD population is still insufficient. The application of biofeedback in HD children has clear limitations. It requires a certain level of cognitive and cooperative ability, and is generally only suitable for children over 4–5 years old, with limited efficacy and compliance in younger age groups. For passive fecal incontinence caused by severe internal sphincter structural injury, simple biofeedback yields limited improvement and often needs to be combined with intervention. The long-term durability of the effect remains controversial, and regular consolidation training is required to maintain the improvement. Therefore, biofeedback should be positioned as an important component of stratified rehabilitation, rather than a universal solution for all postoperative defecation dysfunction.

The core of structured rehabilitation pathway is to clarify the time node and content arrangement of intervention, and rely on the multidisciplinary team cooperation model to provide continuous and individualized management for children from the early postoperative period to adulthood. Studies have shown that postoperative bowel function does not naturally improve with age. A longitudinal follow-up of children undergoing TAPT showed that the incidence of soiling was 52% at both the first follow-up (median age 8.1 years) and the second follow-up (median age 15.4 years), and the incidence of constipation was 20% and 24%, respectively. Given that studies suggest functional deficits may persist, rehabilitation pathways should not be delayed; instead, interventions must be initiated early after surgery (62). The design of rehabilitation pathway should be based on the physiological characteristics and functional requirements at different time points after surgery. One study showed no significant advantage of preoperative daily transanal irrigation (TAI) in preventing postoperative enterocolitis in children with short-segment HD (63). Most patients who undergo a transanal endorectal pull-through (TERP) benefit from botulinum toxin injections. Conservative management can prevent the need for reoperation; however, revision surgery should be reserved exclusively for children who fail to respond to conservative measures (64).

**Figure 1:**
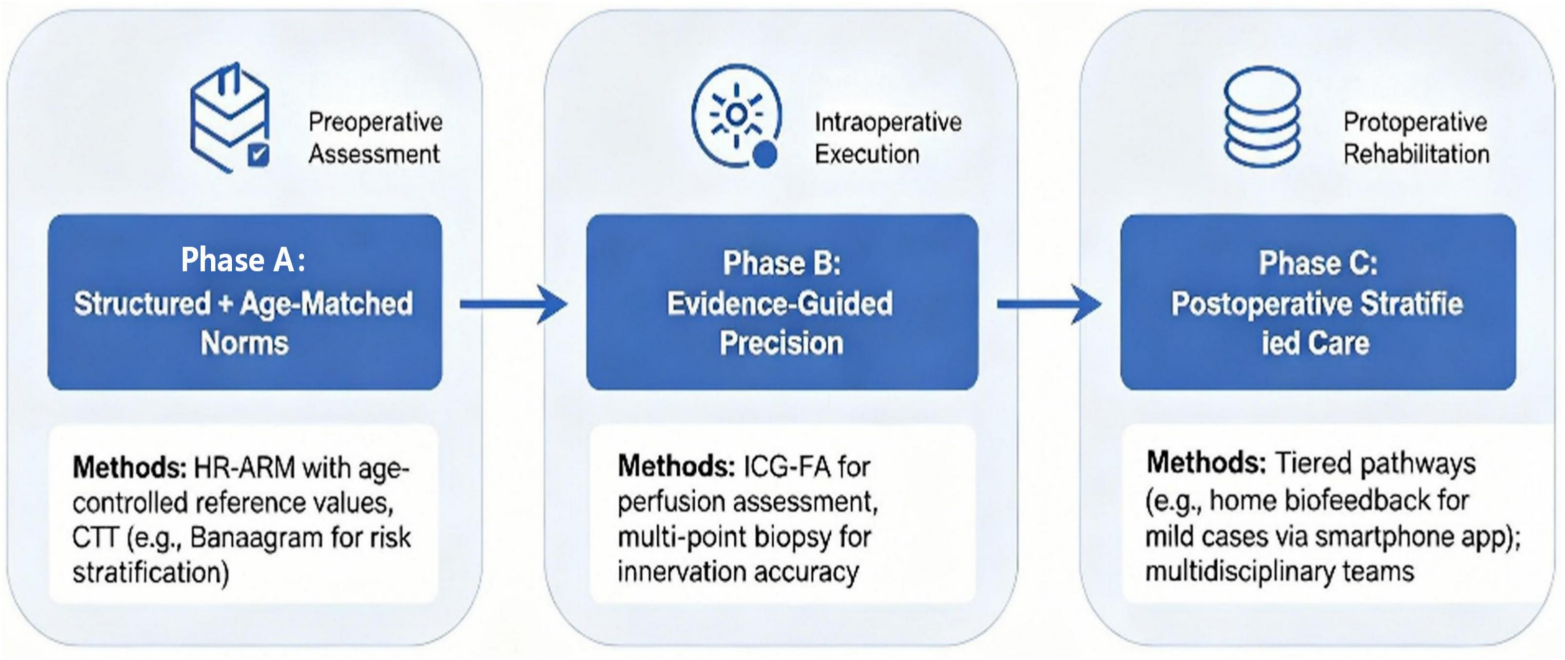
Conceptual framework for paradigm shift in HD management. This framework illustrates the conceptual shift in HD management, moving from subjective, experience-based care towards an objective, prophylactic, and stratified model, requiring standardization and broader technology access.

Postoperative functional monitoring and adjustment should be sustained throughout the child's long-term growth and development. Monitoring should expand beyond isolated clinical outcomes to a multidimensional assessment—including standardized defecation function scores, objective anorectal manometry, quality-of-life measures, and patient/parent-reported experiences. Adjustment strategies should leverage these data to identify high-risk groups and deliver stratified, individualized interventions addressing distinct clinical challenges (Figure 1). For example, for patients with postoperative anastomotic leakage, enterostomy should be performed immediately, and re-pull-out should be selected for repair according to the situation, and the postoperative defecation function score can reach 17.5 ± 1.1 (65). For patients with spillage fecal incontinence caused by residual ganglion cells or transitional zone pathology, re-operation (such as laparoscopic-assisted re-pull-out) is safe and feasible (66). However, comprehensive preoperative evaluation is necessary with the ultimate goal of continuous monitoring and flexible adjustment to maximize the restoration of bowel control, reduce long-term complications, and improve the quality of life comparable to that of their healthy peers.

## 7 Conclusions

From the perspective of the current development trend of radical resection of HD, minimally invasive technology has successfully achieved anatomical accuracy at the surgical level and incision miniaturization, which is undoubtedly a major advance in the history of technology. There is a lack of internationally unified functional evaluation standards. The definitions and assessment tools for defecation dysfunction vary widely across centers, making cross-center comparative research difficult to conduct. The popularization of precision technologies is limited. 3D reconstruction, ICG fluorescence and high-resolution manometry are mostly available in large medical centers, and low-cost, widely applicable solutions are still lacking. The rehabilitation system is underdeveloped. Most centers lack specialized pediatric defecation rehabilitation teams, and intervention is mostly passive treatment after complications, rather than active stratified management.

The core of the whole-cycle functional management paradigm is to take long-term defecation function and quality of life as the ultimate outcome, and to form a closed-loop clinical decision-making system through the organic connection of preoperative, intraoperative and postoperative phases. In the preoperative phase, multi-dimensional assessment including anatomy, function, pathology and genetics is performed to stratify patients into three risk tiers. This risk stratification is not a static classification, but the starting point for individualized decision-making throughout the entire treatment trajectory. It directly guides the selection of surgical approach and the allocation of intraoperative technical resources. In the intraoperative phase, the core principle is to maximize the preservation of functional structures on the premise of ensuring radical resection. For different risk tiers, targeted technical combinations are adopted: for low-risk patients, standard radical resection is sufficient; for intermediate-risk patients, perfusion evaluation and nerve protection techniques are added; for high-risk patients, dual-modal localization and fine dissection techniques should be fully applied to reduce functional injury. In the postoperative phase, rehabilitation intensity and monitoring frequency are matched with the preoperative risk tier. Dynamic re-evaluation is performed at regular follow-up nodes, and the intervention intensity is adjusted flexibly according to the actual functional recovery. Patients with persistent dysfunction are escalated to enhanced intervention, while those with stable good function can be downgraded to routine follow-up, realizing dynamic and precise management throughout the growth and development period. Standardized functional monitoring should be carried out throughout the long-term growth and development of children. The defecation function scoring system, which is widely used in pediatric anorectal malformations, is recommended as the routine evaluation tool. Follow-up is scheduled at 1, 3, 6, and 12 months after surgery, and then annually until adolescence. High-resolution anorectal manometry is performed annually for high-risk patients, and colonic transit test is added for patients with persistent constipation to clarify the etiology of dysfunction. The Pediatric Quality of Life Inventory (PedsQL) is used for school-age children once a year to evaluate the impact of defecation function on physical, psychological and social functions. Caregivers' reports and children's subjective feelings are included in the routine assessment to avoid over-reliance on objective indicators and ignoring actual quality of life.

Innovation and integration should be implemented across three levels: conceptual, instrumental, and systemic. At the instrumental level, artificial intelligence-assisted pathological diagnosis and anorectal manometry analysis may improve the standardization and accuracy of evaluation. Gut microbiome-targeted precise intervention provides a new direction for HAEC prevention. We recommend development of a smartphone-based home biofeedback training module for post-discharge stratified rehabilitation. In addition, the construction of a multicenter big data registry will provide high-level evidence for the optimization of diagnosis and treatment pathways. Explore the optimal window and long-term efficacy of early structured rehabilitation through long-term follow-up studies, and develop evidence-based rehabilitation guidelines. Extend the research scope from HD to a broader range of pediatric gastrointestinal malformations, and explore the general principles of precision minimally invasive functional management.

In conclusion, precision minimally invasive technologies have advanced the surgical management of HD from simple anatomical resection to whole-cycle functional management. The core of this paradigm shift is to take long-term functional outcomes as the guide, integrate multidimensional preoperative assessment, intraoperative precise protection and stratified postoperative rehabilitation, and maximize intestinal function preservation on the premise of ensuring surgical safety. The clinical translation of the functional management concept is still restricted by the lack of unified evaluation standards, limited popularization of precision technologies and imperfect rehabilitation systems. Future research should focus on the construction of standardized evaluation systems, multicenter evidence verification and rehabilitation pathway optimization, so as to provide higher-level evidence for improving the long-term quality of life of children with gastrointestinal malformations.
